# Chondroprotective and anti‐inflammatory effects of amurensin H by regulating TLR4/Syk/NF‐κB signals

**DOI:** 10.1111/jcmm.14893

**Published:** 2019-12-25

**Authors:** Pei Ma, Lifeng Yue, Hui Yang, Yannan Fan, Jinye Bai, Shuyi Li, Jiqiao Yuan, Ziqian Zhang, Chunsuo Yao, Mingbao Lin, Qi Hou

**Affiliations:** ^1^ State Key Laboratory of Bioactive Substance and Function of Natural Medicines Institute of Materia Medica Chinese Academy of Medical Sciences & Peking Union Medical College Beijing China; ^2^ Department of Neology Dongzhimen Hospital Beijing University of Chinese Medicine Beijing China

**Keywords:** amurensin H, NF‐κB, osteoarthritis, Syk, TLR4

## Abstract

The low‐grade, chronic inflammation initiated by TLR4‐triggered innate immune responses has a central role on early osteoarthritis. Amurensin H is a resveratrol dimer with anti‐inflammatory and anti‐apoptotic effects, while its effects on TLR‐4 signals to inhibit osteoarthritis are still unclear. In the present study, treatment with amurensin H for 2 weeks in monosodium iodoacetate‐induced mice significantly slows down cartilage degeneration and inflammation using macroscopic evaluation, haematoxylin and eosin (HE) staining and micro‐magnetic resonance imaging. In IL‐1β‐stimulated rat chondrocytes, amurensin H suppresses the production of inflammatory mediators including nitric oxide, IL‐6, IL‐17, PGE2 and TNF‐α using Greiss and ELISA assay. Amurensin H inhibits matrix degradation via decreasing levels of MMP‐9 and MMP‐13 using Western blot assay, promotes synthesis of type II collagen and glycosaminoglycan using immunostaining and safranin O staining, respectively. Amurensin H inhibits intracellular and mitochondrial reactive oxygen species (ROS) generation, and mitochondrial membrane depolarization using DCFH‐DA, MitoSOX Red and JC‐1 assay as well. IL‐1β stimulates TLR4 activation and Syk phosphorylation in chondrocytes, while amurensin H inhibits TLR4/Syk signals and downstream p65 phosphorylation and translocation in a time and dose‐dependent manner. Together, these results suggest that amurensin H exerts chondroprotective effects by attenuating oxidative stress, inflammation and matrix degradation via the TLR4/Syk/NF‐κB pathway.

**Main topics for review**

1. Amurensin H showed chondroprotective and anti‐inflammatory effects in monosodium iodoacetate‐induced mice and IL‐1β‐stimulated chondrocytes.
2. TLR4 activation was confirmed in IL‐1β‐stimulated chondrocytes, and its downstream signals included Syk, TRAF6 and NF‐κB activation.
3. Amurensin H inhibited TLR4/Syk/NF‐κB signals to block production of inflammatory mediators and matrix degradation.

## 1 INTRODUCTION

Osteoarthritis (OA) is the most common form of chronic arthropathy with cartilage degradation as the universal end‐point, which is a leading cause of pain and disability.^1^, ^2^ Pharmacological treatment for OA such as non‐steroidal anti‐inflammatory drugs cannot change disease course and is associated with adverse effects, and joint replacement is the final choice for patients with end‐stage OA.^3^ Consequently, recent focus is shifting to the identification of novel treatments in early OA to promote joint health.^1^, ^4^

Nowadays, low‐grade, chronic inflammation is considered to have a central role in early OA, and innate immune response is essentially involved in its initiation.^5^ In the early OA joint, many risk factors trigger a progressive local tissue damage and metabolic dysfunction and produce endogenous damage‐associated molecular patterns (DAMPs) which bind to TLR4.^6^ The inflammatory mediators such as IL‐1β, activate TLR4 signalling and trigger the expression of inflammatory factors such as TNF‐α, inducible nitric oxide synthesis (iNOS) and cyclooxygenase 2 (COX­2).^7^, ^8^ This further promotes production of IL‐6, IL‐17, nitric oxide (NO) and PGE2. These inflammatory mediators induce cartilage destruction by activating matrix metalloproteinases (MMPs) such as MMP‐9 and MMP‐13, and inhibiting synthesis of matrix collagen and glycosaminoglycan (GAG), and finally prime a full‐blown inflammatory response and structural damage of the joint.^3^, ^5^, ^9^

The TLR4 downstream signalling events in OA have not been fully elucidated. Different stimuli recruit different adaptor/signalling molecules to TLR4 cytoplasmic domain and trigger various branches of TLR4 signals.^10^ Spleen tyrosine kinase (Syk) has emerged recently as a key effector molecule in TLR4 activation. This signalling seems to be particularly important in innate immune response to DAMPs, which is relevant to chronic inflammatory diseases.^11^, ^12^, ^13^ Recently, basic calcium phosphate crystals, a host‐derived agonist, stimulate IL‐6 secretion through Syk signalling in chondrocytes.^2^ Therefore, understanding the TLR4‐Syk interaction and involved downstream signalling in chondrocytes is important for regulating TLR4 signals and providing therapeutic “entry point” for modifying OA course.

Recent studies suggest natural stilbenes possess anti‐inflammatory and anti‐oxidative properties and inhibit the release of key OA‐related catabolic mediators.^14^ *Vitis amurensis* is a wild grape growing in north‐east and central parts of China, whose leaves and roots are utilized in traditional Chinese medicine for cancer and pain.^15^ Amurensin H (also known as Vam3) isolated from *V. amurensis* is a resveratrol dimer with potential effects on inflammatory diseases including asthma and chronic obstructive pulmonary.^16^, ^17^ Amurensin H is an ATP‐competitive inhibitor of Syk due to the interaction between its ring‐C/D and Syk,^18^ and also, inhibits inflammation via NF‐κB signalling.^19^ Further, we previously have shown that amurensin H inhibits the sodium nitroprusside‐induced chondrocyte apoptosis.^20^ However, for amurensin H, the possible relationship between its chondroprotective and anti‐inflammatory effects, and possible link between its effects on chronic inflammation and TLR4 signals have not been explored in detail.

In the present study, we examined the in vivo and in vitro anti‐inflammatory and chondroprotective effects of amurensin H, and the possible corresponding mechanisms. Results showed that amurensin H inhibited inflammatory and catabolic responses, which was, at least partially, through the inhibition of TLR4‐Syk interaction and NF‐κB‐mediated downstream inflammatory signals.

## 2 MATERIALS AND METHODS

### 2.1 Reagents and animals

**Figure 1:**
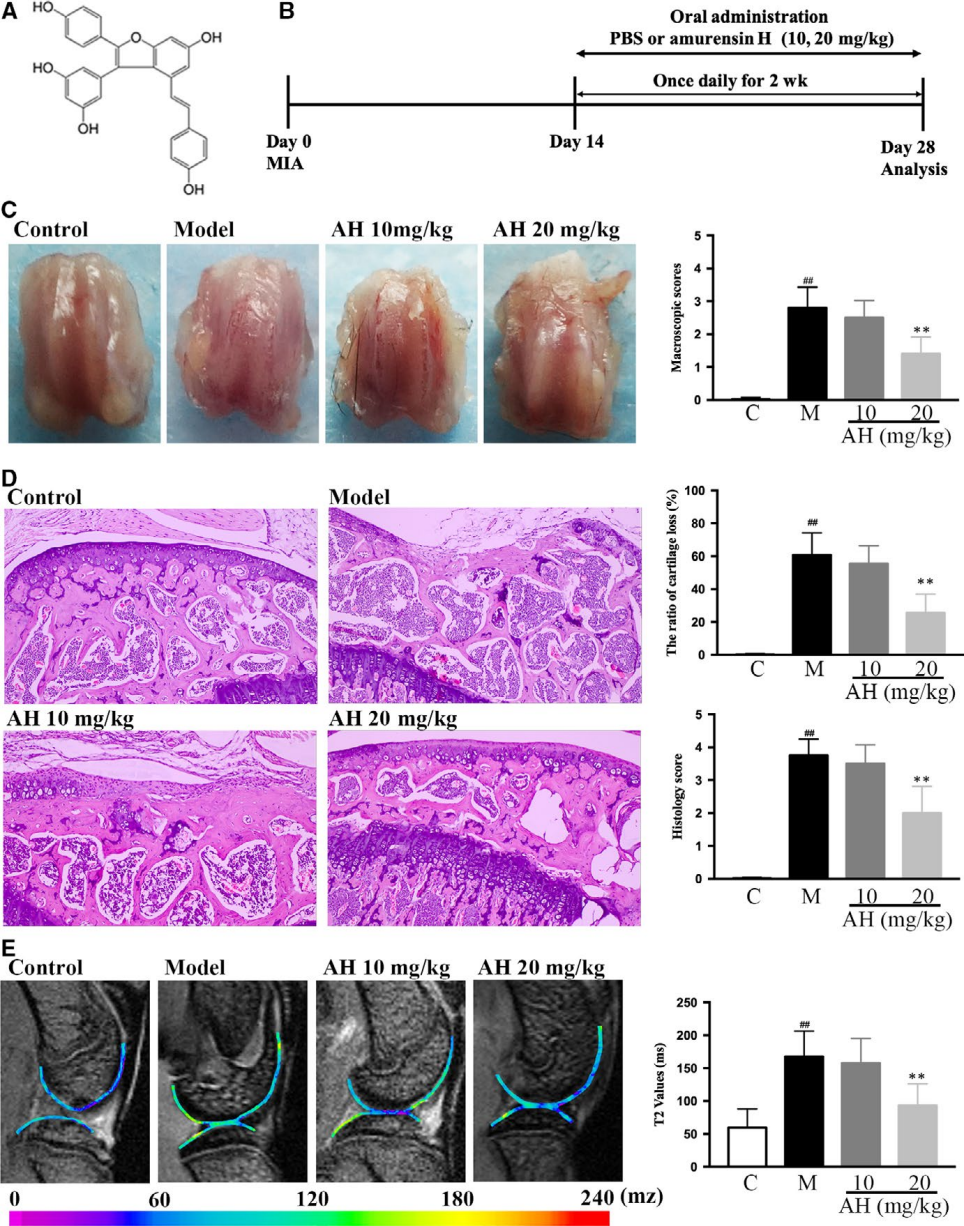
Amurensin H (AH) alleviated monosodium iodoacetate (MIA)‐induced mouse osteoarthritis. (A) Chemical structure of AH, (B) timeline for the development and treatment process of MIA mice, (C) macroscopic appearance and scores of femoral condyles, (D) haematoxylin‐eosin staining (200×), calculated cartilage loss ratio and histological scores of tibial plateau, (E) Micro‐MRI analysis, and calculated T2 values of the region of interest (ROI). n = 3 in each group. #*P* < .05 and ##*P* < .01 vs control group; *P < .05 and **P < .01 vs MIA treated group

Amurensin H was synthesized by Prof. Yao and identified by ESI‐MS and NMR (Figure 1A).^21^ The male SD rats (4‐week‐old) and male C57BL/6 mice (20‐22 g) were purchased from Vital River Laboratory Animal Technology Co. Ltd. All animal experimental procedures were approved by Experimental Animal Care and Use Committee of the Institute of Materia Medica, Chinese Academy of Medical Sciences & Peking Union Medical College (No. 00005784).

### 2.2 Monosodium iodoacetate ‐induced mice model and treatment

Mice were randomly divided into four groups (n = 12): control group, monosodium iodoacetate (MIA) group, and two amurensin H‐treated group (10 and 20 mg/kg/b.wt., respectively). Briefly, mice were anaesthetized. Then, 500 μg MIA (Sigma) was dissolved in sterile saline (0.9%) and injected into the joint capsule. Intra‐articular injection of 10 μL saline was performed as a control. Mice in amurensin H‐treated groups received intra‐gastric administration from day 14, and mice in the rest groups received solvents (Figure 1B).

### 2.3 Macroscopic evaluation

After mice scarification and soft tissue removal, macroscopic evaluation of cartilage on femoral head was performed by two observers using a four‐grade scale (Table S1).

### 2.4 Haematoxylin and eosin staining

Cartilage tissues were fixed by 4% (v/v) paraformaldehyde, decalcified with buffered ethylenediaminetetraacetate (EDTA), dehydrated, paraffin‐embedded and sectioned at 5 μm for H & E staining. A blinded scoring was given by experienced pathologists with a four‐grade scale (Table S2).

### 2.5 Micro‐magnetic resonance imaging

Joints were embedded in Tissue‐Tek OCT Compound (Sakura‐VWR), frozen on dry ice and stored at −80°C. Frozen joints were rehydrated overnight in normal saline at 4°C and scanned in a horizontal position by a 7.0 T micro‐magnetic resonance imaging (micro‐MRI) system (Bruker PharmaScan) with a 23‐mm‐volume coil. The T2 mapping multiple‐spin‐echo sequence was performed (Table S3) and generated with the Bruker ParaVision 6.0 system. The femoral weight‐bearing area was selected as the region of interest, with average T2 relaxation times analysed by a senior musculoskeletal radiologist.

### 2.6 Preparation and treatment of primary rat chondrocytes

Chondrocytes were digested from joints of 2‐3‐week‐old male SD rats.^20^ Briefly, rats were euthanized and immersed into 75% alcohol for 5 minutes. Articular cartilage was resected, digested with 0.25% Trypsin‐EDTA for 30 minutes and collagenase II (Gibco) for 4 hours at 37°C and filtrated through the 200‐mesh strainer. For culture, chondrocytes were seeded at density of 1.8 × 10^4^ cells/cm^2^ in high‐glucose DMEM with 1% penicillin/streptomycin (Solarbio) and 10% FBS (Gibco) in a 37°C and 5% CO_2_ incubator. The phenotype at P1 was identified by typical morphology and immunostaining of type 2 collagen (COL2A1; Abcam) using Inverted Ti‐E fluorescence microscope (Nikon). For experiments, unless otherwise mentioned, chondrocytes (passage 3 to 5) were seeded at density of 3 × 10^4^ cells/cm^2^ and incubated with amurensin H for 2 hours, then, stimulated by 10 ng/mL human IL‐1β (PeproTech) in DMEM with 2.5% FBS. Besides, BAY61‐3606 (Syk inhibitor, Sigma) or TAK‐242 (TLR4 inhibitor, Solarbio) were used to confirm TLR4 signalling.

### 2.7 Cell viability assay and live/dead assay

Chondrocytes grown on 96‐well plates were pre‐treated with amurensin H and stimulated by IL‐1β for 48 hours. Cell viability was determined using MTT (Sigma) assay with OD value measured at 570 nm by a microplate reader.^22^ Chondrocytes grown on 6‐well plates were stained with LIVE/DEAD Cell Imaging Kit (Invitrogen) according to the manufacturer's protocol and analysed by fluorescence microscope. The apoptotic cell death was calculated by dividing the red fluorescent quantification (dead cells) by that of the green fluorescence.

### 2.8 Estimation of levels of nitric oxide, PGE2, IL‐6, IL‐17 and TNF‐α

Chondrocytes were seeded in 96‐well plates, and supernatants were collected after IL‐1β treatment for 48 hours. NO level was estimated using Greiss assay.^23^ The levels of IL‐6, IL‐17, TNF‐α and PGE2 were estimated using commercial ELISA kits (R&D Systems) according to the manufacturer's protocol with OD values measured at 450 and 570 nm.

### 2.9 Estimation of glycosaminoglycan content

Chondrocytes at P1 were seeded on coverslips onside 6‐well plates (2 × 10^4^/well) and treated by IL‐1β for 48 hours, then fixed by 4% (v/v) paraformaldehyde, stained with Safranin O (SO, Sigma) for histological evaluation of cell morphology and GAG production. Chondrocytes seeded in 6‐well plates were treated by IL‐1β for 96 hours, digested by proteinase K (Solarbio). The enzymatic hydrolysates were centrifuged. DNA content was measured by a spectrofluorometer using Hoechst 33258 dye at 460 nm (emission) and 360 nm (excitation). A series of diluted chondrocytes (1 × 10^6^ cells) were used as a control.^24^ The total intracellular sGAG secretion was qualitied spectrophotometrically at 525 nm using 1,9‐dimethylmethylene blue dye (Sigma) with chondroitin sulphate (Sigma) as a standard, and normalized to the total DNA content.

### 2.10 Measurement of intracellular reactive oxygen species, mitochondrial ROS and mitochondrial membrane potential (ΔѰM)

Chondrocytes were seeded in 6‐well plate (2 × 10^4^/well, image analysis) or 96‐well plate, pre‐treated with amurensin H, stimulated with IL‐1β for 6 hours and labelled with DCFH‐DA (10 μmol/L, Sigma), MitoSOX Red (5 μmol/L, Invitrogen) or JC‐1 (5 μg/mL; Beyotime, Nanjing, China) for 30, 10 or 20 minutes at 37°C, respectively. Fluorescent signals were observed using fluorescence microscopy and fluorescence plate reader, respectively (Table S4). Loss of ΔѰM was calculated as the fluorescence ratio of red (aggregate form) to green (monomer form).

### 2.11 Western immunoblotting

Chondrocytes were treated on 6 cm dish, washed with cold PBS, lysis with RIPA (Solarbio) containing a protease inhibitor mixture.^25^ A total of 30 μg protein were resolved by 10% SDS‐PAGE, transferred to a PVDF membrane, blocked with 5% milk, incubated with antibodies against COX‐2, iNOS, MMP‐9, MMP‐13, TLR4, TRAF‐6, Syk, p‐Syk, p65, p‐p65 and β‐actin (1:1000; Cell Signaling Technology) at 4°C overnight and hybridized with HRP‐conjugated secondary antibody for 1 hours. The immunoreactive bands were visualized using an ECL system (CLINX). The relative intensities of bands were quantified using Image J.

### 2.12 Immunofluorescence assay

Chondrocytes were treated in 6‐well plate (2 × 10^4^/well), fixed by 4% (v/v) paraformaldehyde, permeabilized using 0.5% Triton X‐100, blocked with 3% goat serum, incubated overnight at 4°C with antibodies against COL2A1, TLR‐4 (Abcam) and p65, and followed by incubation with antimouse or antirabbit Alexa Fluor 594 secondary antibody (Life Technologies) for 30 minutes. Cells were counterstained with DAPI for 5 minutes and visualized by fluorescence microscope, with fluorescence intensity measured by Image J.

### 2.13 Statistical analyses

Values were presented as Mean ± SD. The statistically significant difference between experimental groups and control was analysed via one‐way ANOVA followed by post hoc Tukey test. *P* < .05 was considered as statistically significant.

## 3 RESULTS

### 3.1 Amurensin H alleviates the progression of MIA‐induced OA in mouse model

Intra‐articular injection of MIA disrupts cellular glycolysis and induces cell death, which triggers a local acute inflammation and subsequent cartilage degeneration. It is a widely used method to mimic structural and functional changes of human OA.^26^ Here, normal cartilage with a smooth surface on the femoral condyles was macroscopically observed in the control group, while significantly thinning cartilage was observed in the model group (*P* < .01, Figure 1C). Twenty mg/kg of amurensin H significantly alleviated bone wear, compared to the model group (*P* < .01).

Histological examinations also showed significant cartilage fibrillation, cartilage loss up to 60 ± 13.4%, subchondral bone erosion, inflammation and pannus formation in the model vs the control group (*P* < .01), and 20 mg/kg of amurensin H significantly alleviated this trend with 25.5 ± 11.4% of cartilage loss (*P* < .01, Figure 1D).

The recent development of high field strength imaging and functional MRI T2 mapping enables accurate morphologic assessment of early degenerative changes in chondral lesions, provides quantitative information about GAG and collagen variations in the extracellular matrix (ECM) and assesses changes in the compressive stiffness of the articular cartilage.^27^ Significantly elevated signal intensity was observed in the T2 maps of cartilage in the model group compared with that in control group (167.8 ± 35.3 ms vs 59.5 ± 26.3 ms, *P* < .01, Figure 1E). The average T2 values in 20 mg/kg of amurensin H‐treated group were 93.23 ± 30.3 ms, significantly lower than those of the model group (*P* < .01). Taken together, amurensin H significant slowed down OA course in MIA‐induced mouse model.

### 3.2 Amurensin H inhibits IL‐1β‐induced inflammatory mediators in chondrocytes

To further investigate the in vitro chondroprotective effects of amurensin H, primary chondrocytes were isolated from rat knee and maintained typical morphology and high expression of COL2A1 at P1 (Figure S1). Amurensin H treatment (2‐8 μmol/L, 48 hours) had no obvious effect on cell viability (Figure S2). Ten ng/mL IL‐1β induced a maximal production of NO and IL‐6 after incubation for 48 hours (Figure S3).

**Figure 2:**
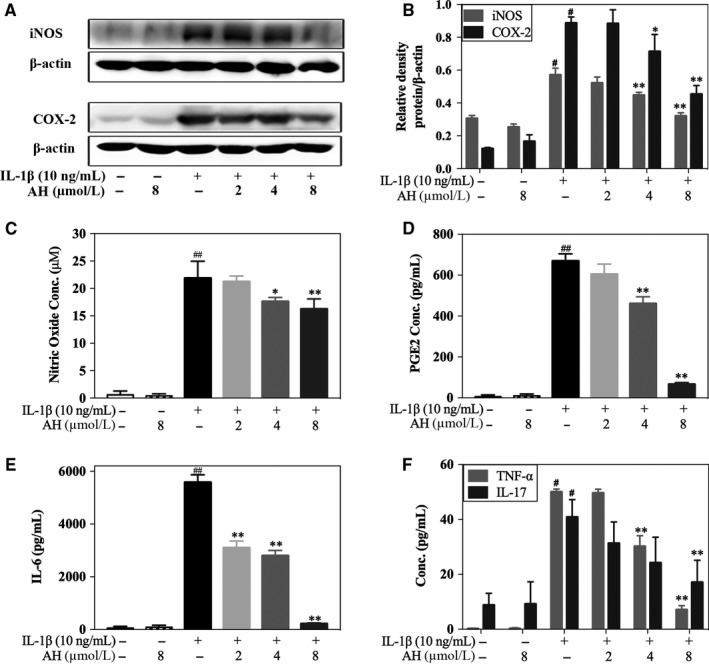
Amurensin H (AH) inhibited IL‐1β‐induced inflammatory mediators in rat chondrocytes. Chondrocytes were treated with IL‐1β for 24 h (A, B) or 48 h (C‐F) after pre‐incubation with AH for 2 h (n = 6). (A‐C) Cytosolic iNOS expression and NO level were attenuated by AH treatment. (A, B and D) Cytosolic COX‐2 expression and PGE2 level were attenuated by AH treatment. (E, F) Levels of IL‐6, TNF‐α and IL‐17 were decreased after AH treatment. β‐Actin was used as a control for equal loading. #*P* < .05 and ##*P* < .01 vs control group; *P < .05 and **P < .01 vs IL‐1β‐treated group

The significantly elevated NO levels and iNOS expression induced by IL‐1β were decreased significantly by pre‐treatment with 4 μmol/L and 8 μmol/L of amurensin H (*P* < .05 or *P* < .01) (Figure 2A‐C). The increased PGE2 levels and COX‐2 expression were also significantly alleviated after pre‐treatment with 4 μmol/L and 8 μmol/L of amurensin H (*P* < .05 or *P* < .01) (Figure 2A,B,D). Besides, IL‐1β induced a significant secretion of IL‐6, IL‐17 and TNF‐α (*P* < .01 vs control), and amurensin H blocked this trend in a dose‐dependent manner (*P* < .01 at 8 μmol/L, Figure 2E,F). Taken together, amurensin H exhibited favourable biocompatibility and exhibited potent anti‐inflammatory effects in pathological chondrocytes.

### 3.3 Amurensin H inhibits IL‐1β‐induced oxidative stress in chondrocytes

**Figure 3:**
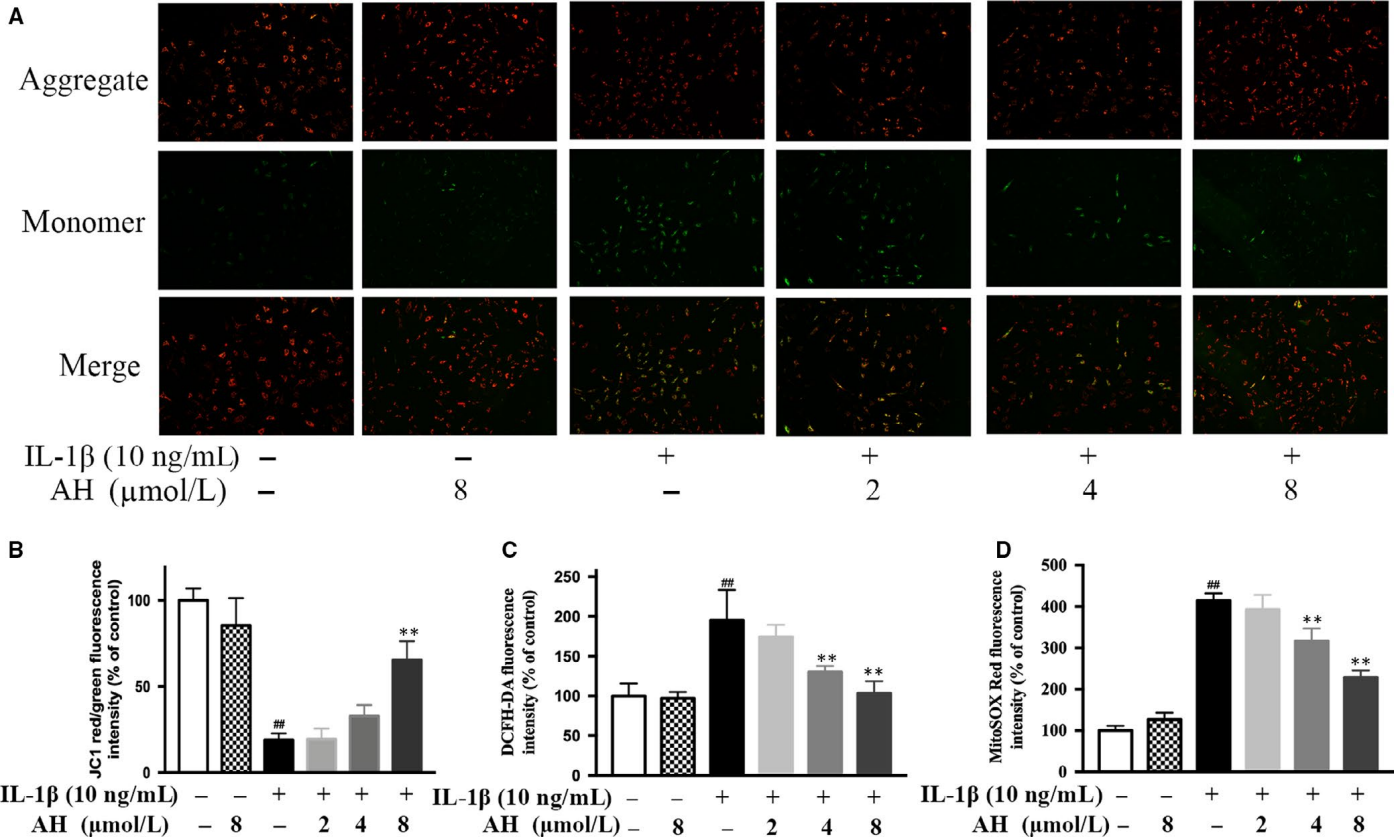
Amurensin H (AH) inhibited IL‐1β‐induced ROS generation in rat chondrocytes. (A, B) The mitochondrial membrane potential was measured using JC‐1. The total and mitochondrial ROS were measured using DCFH‐DA (C) and MitoSOX Red (D). n = 6 in each group. #*P* < .05 and ##*P* < .01 vs control group; *P < .05 and **P < .01 vs IL‐1β‐treated group

The mitochondrial membrane depolarization and dysfunction were evaluated by change in ΔѰM. IL‐1β significantly induced mitochondrial damage (20.3 ± 3.6% of control), which could be significantly reversed by 8 μmol/L of amurensin H (57.5 ± 4.9% of control, *P* < .01, Figure 3A,B). Compared to normal chondrocytes, the total and mitochondrial ROS were significantly increased in IL‐1β‐stimulated chondrocytes (195.1 ± 31.3% and 414.3 ± 16.1% of control, respectively, *P* < .01), whereas amurensin H significantly suppressed their generation in a dose‐dependent manner (103.4 ± 12.5% and 220.4 ± 10.0% of control, respectively, at 8 μmol/L, *P* < .01, Figure 3C,D). Taken together, amurensin H inhibited oxidative stress and mitochondrial damage in pathological chondrocytes.

### 3.4 Amurensin H inhibits IL‐1β‐induced matrix degradation in chondrocytes

**Figure 4:**
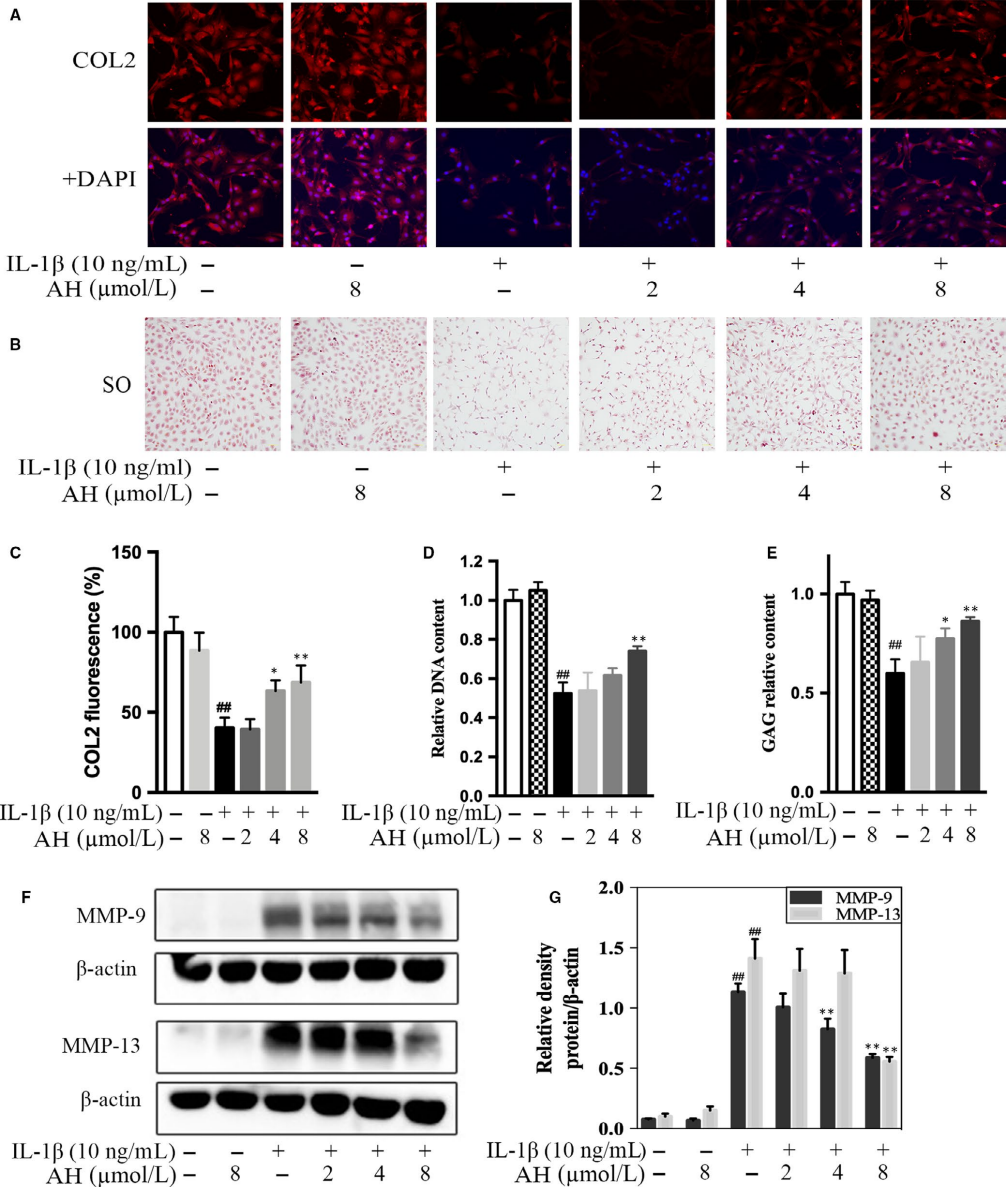
Amurensin H (AH) inhibited IL‐1β‐induced matrix degradation in rat chondrocytes. Chondrocytes were treated with IL‐1β for 48 h (A‐C, F, G) or 96 h (D, E) after pre‐incubation with AH for 2 h (n = 6). (A‐C) Immunofluorescent and safranin O staining images revealed increased production of COL2 (200×) and GAG (100×) after AH treatment. (D, E) Increased cytosolic production of GAG and relative quantification of DNA content were observed after AH treatment. (F, G) Levels of MMP‐9 and MMP‐13 were decreased after AH treatment. β‐Actin was used as a control for equal loading. #*P* < .05 and ##*P* < .01 vs control group; *P < .05 and **P < .01 vs IL‐1β‐treated group

The COL2 and GAG are major ECM components. Eight μmol/L of amurensin H significantly rescued IL‐1β induced down‐regulation of COL2A1 (68.6 ± 8.6% vs 40.3 ± 5.2%, *P* < .01, Figure 4A,C). Amurensin H also promoted GAG production in a dose‐dependent manner as the SO staining was more intense than that of the model (Figure 4B). The results of biochemical GAG secretion assays were similar, as 8 μmol/L of amurensin H significantly increased GAG level at the fourth day (86.1 ± 1.8% vs 59.9 ± 5.8%, *P* < .01, Figure 4D,E).

Levels of MMP‐9 and MMP‐13 were significantly increased in IL‐1β‐stimulated chondrocytes, whereas amurensin H treatment significantly suppressed their expression in a dose‐dependent manner (Figure 4F,G). Altogether, amurensin H down‐regulated the expression of catabolic factors and up‐regulated the expression of anabolic factors in pathological chondrocytes.

### 3.5 Amurensin H regulates IL‐1β‐induced TLR4‐Syk interaction in chondrocytes

**Figure 5:**
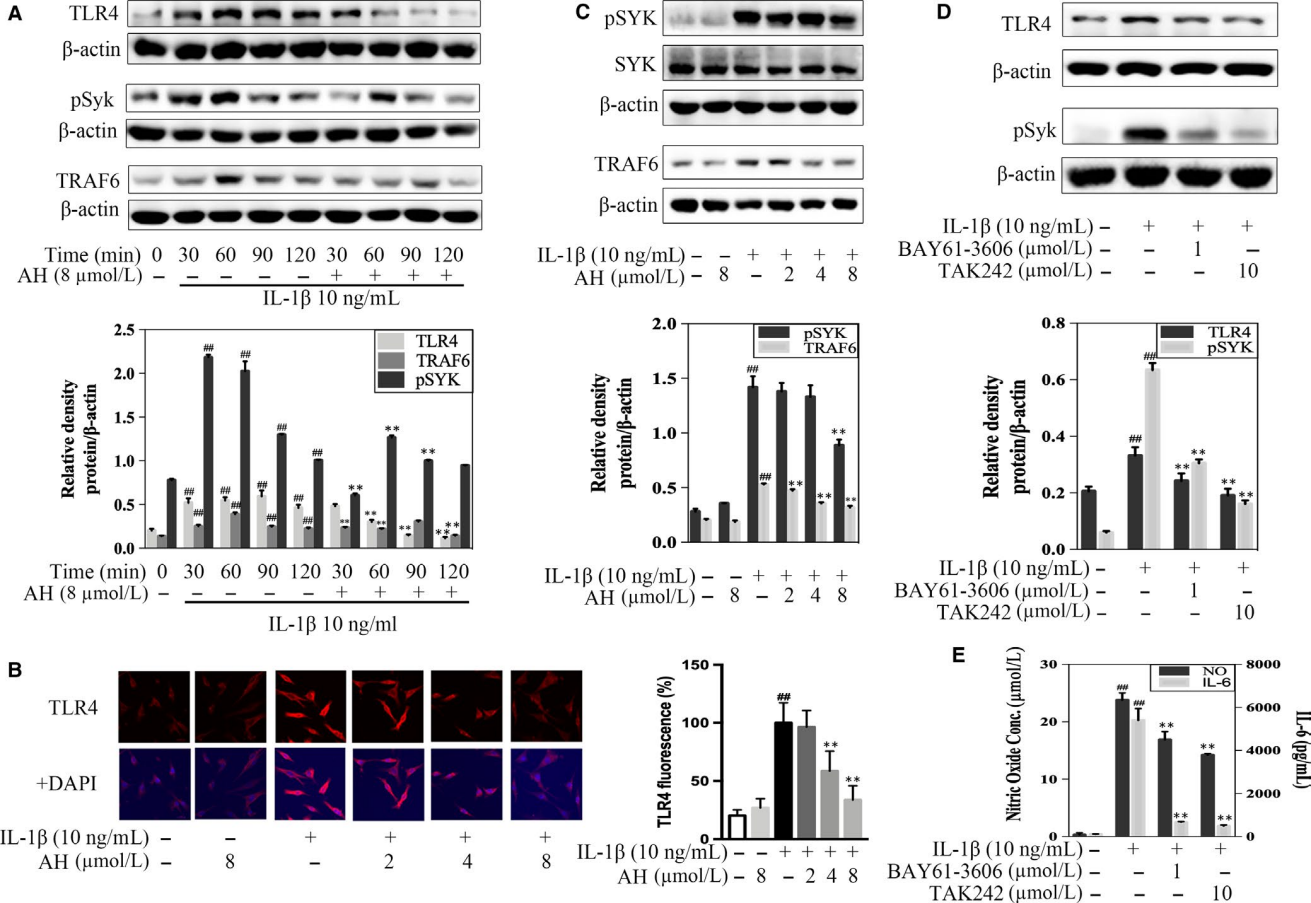
Amurensin H (AH) inhibited TLR4‐triggered signals in rat chondrocytes. Chondrocytes were treated with IL‐1β for indicated time‐points (A), 1 h (B‐D) or 48 h (E) after pre‐incubation with AH or inhibitors for 2 h (n = 3). (A) AH inhibited protein expressions of TLR4, p‐Syk and TRAF6 in a time‐dependent manner. (B, C) AH inhibited protein expressions of TLR4 (200×), p‐Syk and TRAF6 in a dose‐dependent manner. (D) Protein expressions of TLR4 and p‐Syk were attenuated by TAK242 and BAY61‐3606. (E) Inhibition of TLR4 and Syk alleviated levels of nitric oxide and IL‐6. β‐Actin was used as a control for equal loading. #*P* < .05 and ##*P* < .01 vs control group; *P < .05 and **P < .01 vs IL‐1β‐treated group

We next determined the molecular mechanism responsible for chondroprotective effects of amurensin H. We firstly measured TLR4, Syk and TRAF6 expression and observed a significant increase within 120 minutes after stimulation (*P* < .01, Figure 5A). The above increased expression or phosphorylation was blocked by 8 μmol/L of amurensin H treatment. Besides, amurensin H inhibited TLR4, TRAF6 expression and Syk phosphorylation in a dose‐dependent manner as well (Figure 5B,C). We further confirmed the role of TLR4‐Syk interaction using TLR4 and Syk inhibitor, TAK242 and BAY61‐3606, respectively. Results showed that TAK242 and BAY61‐3606 significantly abrogated the secretion of nitric oxide and IL‐6 (*P* < .01, Figure 5E). Besides, increased expression of TLR4 and phosphorylation of Syk was attenuated more obviously by TAK242 (Figure 5D).

### 3.6 Amurensin H regulates IL‐1β‐induced translocation and phosphorylation of p65 in chondrocytes

**Figure 6:**
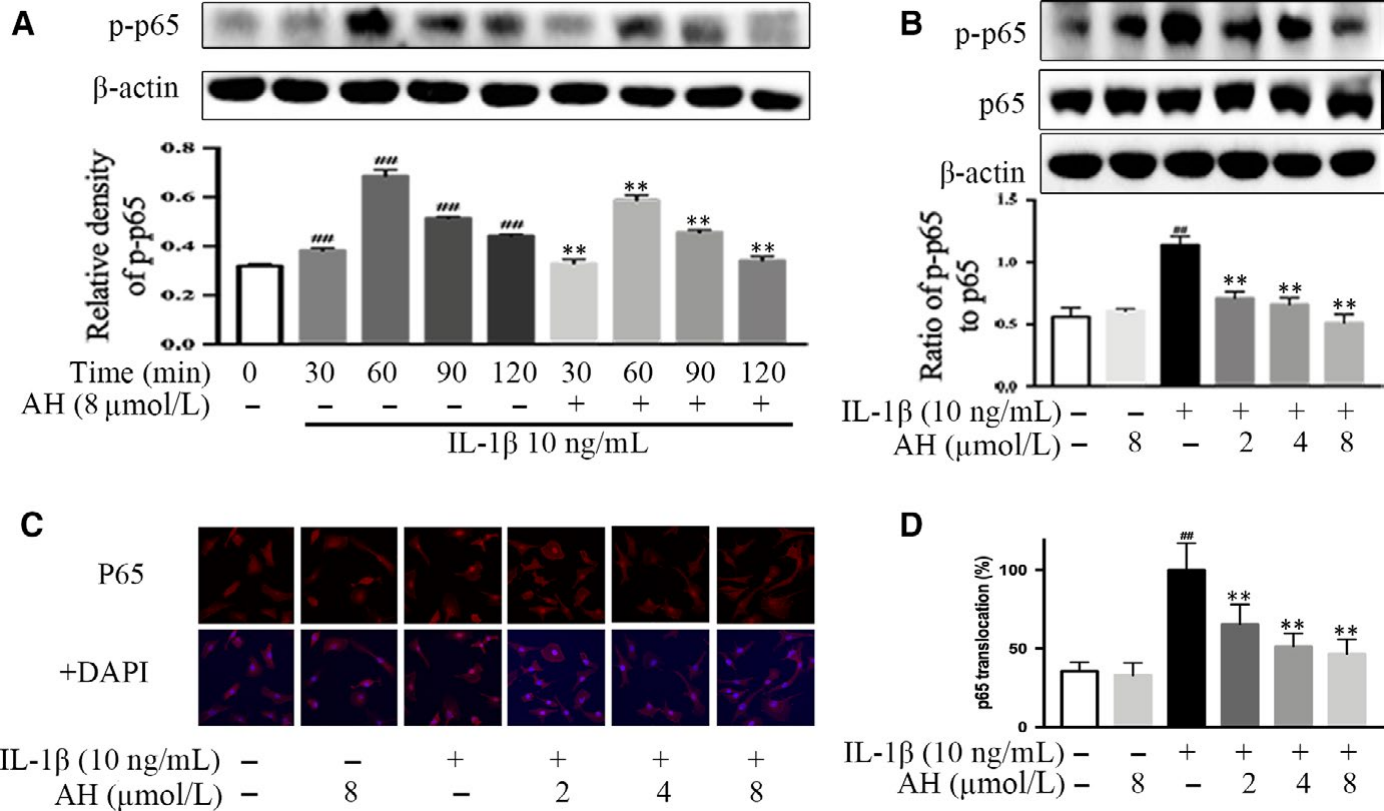
Amurensin H (AH) inhibited downstream pro‐inflammatory transcription factor NF‐κB in rat chondrocytes. Chondrocytes were treated with IL‐1β for indicated time‐points (A) or 1 h (B, C) after pre‐incubation with AH for 2 h (n = 3). (A) AH inhibited p65 phosphorylation in a time‐dependent manner. (B, C, D) AH inhibited p65 phosphorylation and p65 (F, 200×) translocation in a dose‐dependent manner. β‐Actin was used as a control for equal loading. #*P* < .05 and ##*P* < .01 vs control group; *P < .05 and **P < .01 vs IL‐1β‐treated group

The effects of amurensin H on the activation of NF‐κB p65 were examined, and results showed a significant increasing expression of p‐p65 within 120 minutes after stimulation, which was blocked by 8 μmol/L of amurensin H treatment (*P* < .01, Figure 6A). Corroborating the time‐dependent manner, there was a dose‐dependent inhibition in the phosphorylation and nuclear translocation of p65 in amurensin H‐treated chondrocytes (*P* < .01, Figure 6B‐D).

## 4 DISCUSSION

**Figure 7:**
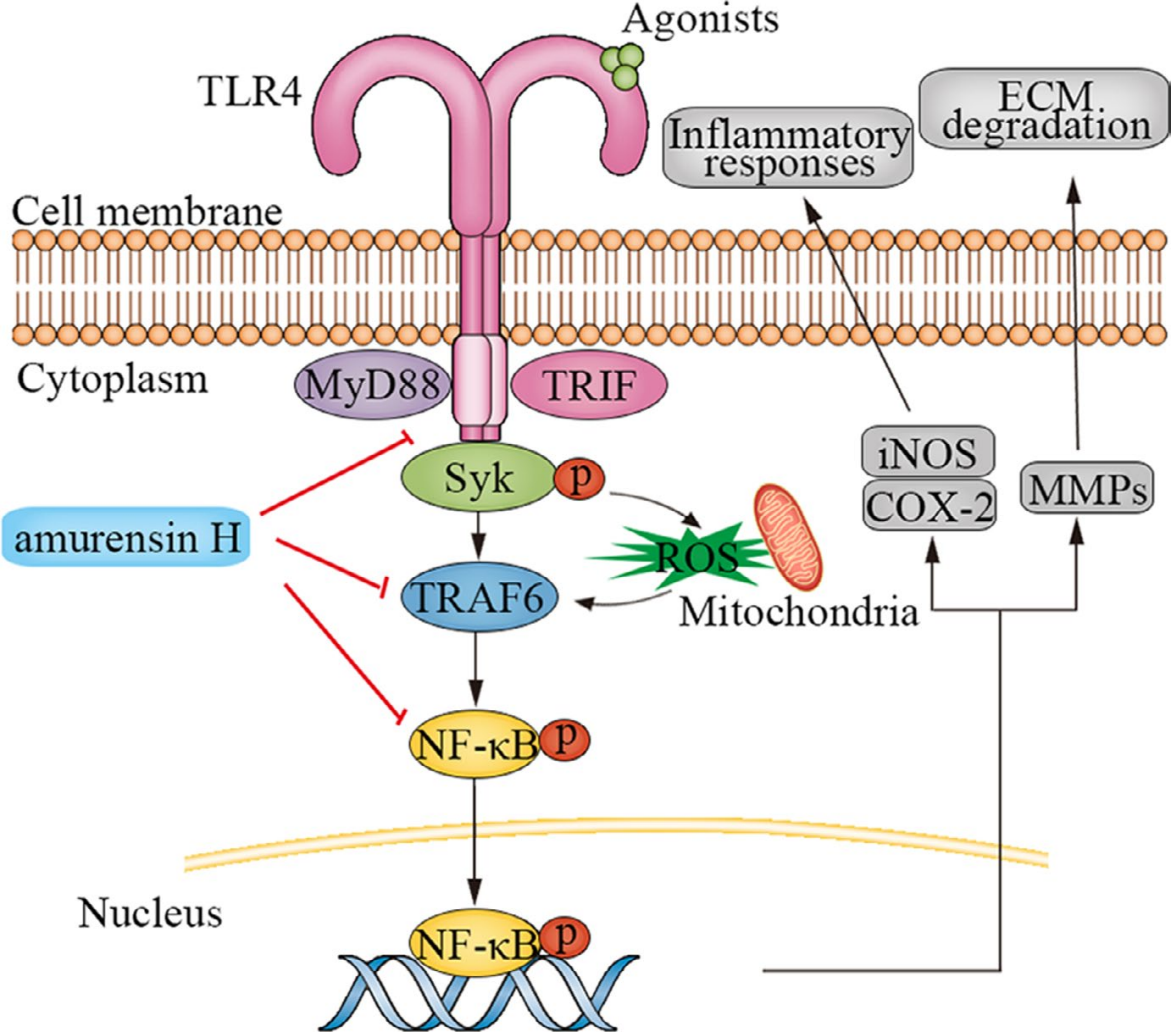
A schematic diagram of proposed mechanism of amurensin H on TLR4/Syk/NF‐κB signals in rat chondrocytes. Amurensin H treatment negatively regulated TLR4 activation, Syk phosphorylation, TRAF6 activation, and phosphorylation and translocation of p65 in IL‐1β‐stimulated chondrocytes, thereby inhibiting inflammation and matrix degradation

TLR4 is the main regulators of innate immunity activated in OA, while little is known for its downstream signalling involved in OA. This study is undertaken to test the hypothesis that TLR4/Syk/NF‐κB signals are initiated in early OA and providing therapeutic “entry point” for modifying OA. We found that amurensin H alleviated cartilage lesions in MIA‐induced OA mice and protected chondrocytes against IL‐1β‐induced inflammation and matrix degradation via TLR4/Syk/NF‐κB signalling. Our results indicate that TLR4/Syk/NF‐κB signalling is activated in inflammatory chondrocytes, and amurensin H is pharmacologically effective against OA pathogenic events (Figure 7).

Amurensin H is a stilbene with in vitro and in vivo anti‐inflammatory effects. Amurensin H alleviates inflammatory response via IL‐1β signals in macrophages.^28^ Here, amurensin H also significantly attenuates IL‐1β‐induced expression of COX‐2, PGE2, iNOS, NO, TNF‐α, IL‐6 and IL‐17 in chondrocytes. IL‐1 and TNF‐α increase the synthesis of PGE2 via activating COX‐2, up‐regulate the production of NO via stimulating iNOS and also promote inflammatory cytokines such as IL‐6 and IL‐17, all of which contribute to cartilage destruction and promote OA progression.^7^, ^8^, ^29^ Therefore, our results show that amurensin H could inhibit low‐grade, chronic inflammation in chondrocyte to block early OA.

Amurensin H alleviates inflammation‐induced ECM destruction. In asthmatic‐like mice, amurensin H inhibits MMP‐9 and ICAM‐1 activities.^30^ Here, amurensin H promotes ECM anabolism via affecting GAGs and COL2A1 deposition, inhibits ECM catabolism via affecting MMP‐9 and MMP‐13 expression in IL‐1β‐stimulated chondrocyte. As matrix degradation is the hallmark of OA,^31^ amurensin H is a potential candidate for disease‐modifying osteoarthritis drugs.

Like many other ageing disease, oxidative stress is implicated in inflammatory signalling cascade in OA. The oxidative stress not only promotes related inflammatory process, but induces mitochondrial dysfunction, chondrocyte apoptosis and finally ECM degradation.^32^ Amurensin H significantly suppressed IL‐1β‐mediated intracellular and mitochondrial ROS generation, as well as the elevation of ΔѰM in a dose‐dependent manner. The anti‐oxidative stress effect of amurensin H is important for its anti‐inflammatory effects, as amurensin H also inhibits induced oxidative stress and mitochondrial dysfunction to decrease apoptosis and autophagy in inflammatory bronchial epithelial cells.^17^

Now that low‐grade, chronic inflammation has a central role in early OA, what is the key signalling event to initiate it? A growing body of evidence suggests that TLR4 is activated by DAMPs, and highly expressed in cartilage lesion, thus induces inflammatory and catabolic responses, which is important for OA course.^3^ Recent studies show that TLR4 signals divide into several branches due to different adaptor/signalling molecules, among which Syk is a key effector activated by DAMPs in chronic inflammatory diseases.^11^, ^12^, ^13^ However, the TLR4‐Syk interaction and involved downstream signalling in OA have not been explored in detail. Our studies show that IL‐1β induces TLR4 activation and Syk phosphorylation in chondrocytes, interrupting them separately by inhibitors attenuates inflammation, thus TLR4/Syk is an important innate immune response, which triggers chronic, low‐grade inflammation in OA. Amurensin H blocks TLR4/Syk activation, which attributes to its anti‐inflammatory and chondroprotective effects.

TLR4‐Syk interaction is complicated due to possible crosstalk with different branches of TLR4 signals. In macrophages stimulated by host‐derived mmLDL, Syk links TLR4 activation with intracellular PLC/Nox2 signalling and subsequent generation of ROS and inflammatory cytokines.^11^ In macrophages stimulated by LPS, endocytosis of TLR4 activates downstream Syk/TRIF/IRF3 signalling and delayed NF‐κB activation.^33^ In human proximal tubular epithelial cells, high glucose induces an immediate ROS‐dependent extracellular release of HMGB‐1 which activates TLR4/MyD88/Syk/NF‐κB.^12^ In mice with retinal ischaemia/ reperfusion injury, Syk and NF‐κB are key molecules in TLR4 downstream signalling.^13^ Thus, NF‐κB is an important downstream transcriptional factor of TLR/Syk signalling, which induces pro‐inflammatory cytokine production.^3^ Considering TRAF6 regulates NF‐κB for its entry into the nucleus in TLR4 signalling,^34^ we evaluate expression of TRAF6 and NF‐κB. Results show that IL‐1β leads to TRAF6 and NF‐κB activation, which is inhibited by amurensin H. Therefore, amurensin H inhibits inflammation via regulating TLR4/Syk/NF‐κB signalling in chondrocytes.

Our results confirm that innate immune response is involved in early OA, trigger chronic low‐grade inflammation and subsequent ECM degradation, which supports potential therapeutic targets to slow or change disease course in early OA. Amurensin H has in vivo and in vitro anti‐inflammatory and chondroprotective effects and inhibits TLR4/Syk/NF‐κB signalling in chondrocytes, suggesting that amurensin H could be a potential candidate for disease‐modifying osteoarthritis drugs.

## CONFLICT OF INTEREST

The authors have no conflict of interest to declare.

## AUTHOR CONTRIBUTION

Pei Ma, Mingbao Lin and Qi Hou designed the research. Pei Ma, Lifeng Yue, and Jinye Bai performed and analysed animal experiments. Pei Ma, Hui Yang, Yannan Fan, Shuyi Li, Jiqiao Yuan, Ziqian Zhang performed and analysed cell experiments. Pei Ma prepared the manuscript. Chunsuo Yao provided amurensin H. Mingbao Lin and Qi Hou revised the manuscript content.

## Data Availability

The data that support the findings of this study are available from the corresponding author upon reasonable request.
