# Supplementary figures and images for: Chondroprotective and anti‐inflammatory effects of amurensin H by regulating TLR4/Syk/NF‐κB signals
Source: J Cell Mol Med. 2019 Dec 25;24(2):1958–68. doi: 10.1111/jcmm.14893 (PMC6991675; doi:10.1111/jcmm.14893)

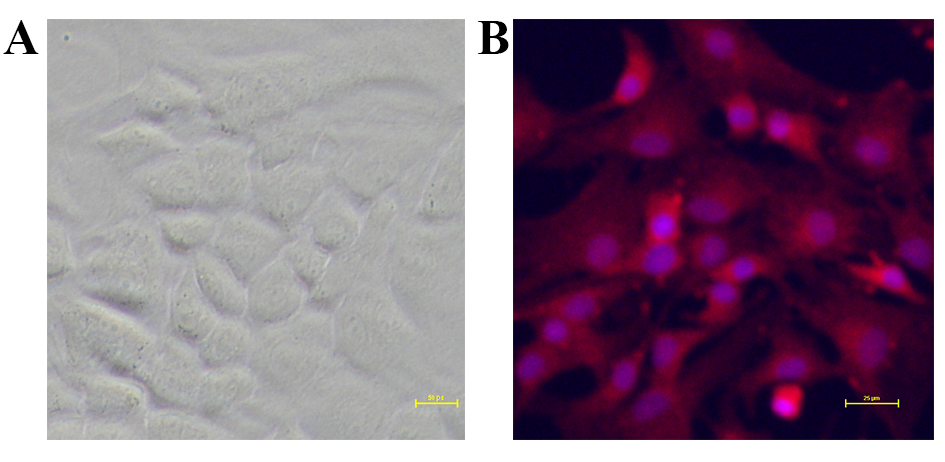

Supplement: Supplementary file 1 [file JCMM-24-1958-s001.tif]
