# Supplementary material for: Chondroprotective and anti‐inflammatory effects of amurensin H by regulating TLR4/Syk/NF‐κB signals
Source: J Cell Mol Med. 2019 Dec 25;24(2):1958–68. doi: 10.1111/jcmm.14893 (PMC6991675; doi:10.1111/jcmm.14893)
Supplement: Supplementary file 4 [file JCMM-24-1958-s004.doc]

**Supporting Information**

Table S1 Scoring system for macroscopic evaluation

| score | criteria |
| --- | --- |
| 0 | normal-appearing surface |
| 1 | minimal fibrillation or a slight yellow discoloration of the surface |
| 2 | erosion extending into the superficial or middle layers only |
| 3 | erosion extending into the deep layers |
| 4 | erosion extending to the subchondral bone |

Table S2 Scoring system for H&E staining

| score | cartilage | | | subchondral bone | inflammation | pannus |
| --- | --- | --- | --- | --- | --- | --- |
|  | loss ratio | fibrillation | erosion |  |  |  |
| 0 | - | - | - | - | - | - |
| 1 | - | - | + | - | + | - |
| 2 | + | + | ++ | - | + | - |
| 3 | ++ | + | +++ | - | + | - |
| 4 | +++ | + | +++ | + | ++ | + |

Loss ratio: “-” no loss, “+” <25%, “++” 25% ~ 50%, “+++” > 50%;

Fibrillation: “-” no fibrillation, “+” fibrillation;

Erosion: “-” no change, “+” thinning, “++” erosion, “+++” erosion down to the subchondral bone;

Subchondral bone: “-” no erosion, “+” erosion;

Inflammation: “-” no, “+” slight, “++” moderate;

Pannus: “-” no, “+” pannus formation;

Table S3 Condition for T2 mapping multiple-spin-echo sequence

| index | value |
| --- | --- |
| epetition time (TR, ms) | 2000 |
| echo times (TEs, ms) | 11.0, 22.9, 34.8, 46.7, 58.6, 70.5 |
| flip angle (FA, degree) | 90 |
| field of view (FOV, mm) | 15 × 15 |
| slice thickness (ST, mm) | 1 |
| acquisition time (TA, min) | 8.5 |
| matrix size (pixels) | 256 × 256 |
| slices (count) | 25 |

Table S4 Excitation and emission wavelength for fluorescence detection

| assay | excitation (nm) | emission (nm) |
| --- | --- | --- |
| DCFH-DA | 488 | 525 |
| MitoSOX Red | 510 | 580 |
| JC-1 | 585 | 590 |
|  | 514 | 529 |

Figure S1 Identification of isolated chondrocytes. (A) Chondrocytes at P1 typically had a round or polygonal shape with granular cytoplasm. (B) Chondrocytes at P1 maintained phenotype with high expression of COL2A1.

Figure S2 Amurensin H (AH) did not affect cell viability or cell death (n=6). (A) AH treatment for 48h did not affect cell viability. (B) AH treatment alleviated cell death induced by IL-1β for 48h.

Figure S3 Generation of IL-1β-induced inflammatory mediators (n=6). The levels of nitric oxide (A) or IL-6 (B) increased in a time dependent manner. The level of nitric oxide increased in a dose dependent manner (C), and cell viability was affected (D).
